# The value of transcranial Doppler monitoring of cerebral blood flow changes during carotid endarterectomy performed under regional anesthesia – A case series

**DOI:** 10.1515/tnsci-2022-0257

**Published:** 2022-12-16

**Authors:** Zoltán Gyöngyösi, Orsolya Farkas, Lóránd Papp, Fruzsina Bodnár, Tamás Végh, Béla Fülesdi

**Affiliations:** Department of Anesthesiology and Intensive Care, University of Debrecen, H-4030, Nagyerdei krt. 98, Debrecen, Hungary; Department of Surgery, Faculty of Medicine, University of Debrecen, Debrecen, Hungary; Outcomes Research Consortium, Cleveland, OH, USA

**Keywords:** carotid endarterectomy, regional anesthesia, transcranial Doppler monitoring

## Abstract

Recent evidence suggests no difference between patient outcomes when carotid endarterectomies (CEAs) are performed under general or regional anesthesia. However, for detecting the need for a shunt, general anesthesia has the drawback of monitoring needs in the intraoperative setting. In the present study, we attempted to perform intraoperative transcranial Doppler (TCD) monitoring for CEAs performed under intermediate plexus block to describe cerebral hemodynamic changes during different phases of the procedure.

**Patients and methods:**

Patients with unilateral hemodynamically significant carotid stenosis scheduled for elective CEAs were included. Ultrasound-guided intermediate plexus block was used for regional anesthesia. TCD monitoring of the middle cerebral artery mean blood flow velocity (MCAV) was performed throughout the procedure. MCAVs were offline analyzed during different phases of CEA: (1) resting state, before regional block, (2) after block, before incision, (3) before cross-clamp, (4) after cross-clamp, (5) 5 min after cross-clamp, (6) 10 min after cross-clamp, (7) after declamping, and (8) during the postoperative period (4–6 h).

**Results:**

Shunt insertion based on the deterioration of neurological symptoms after cross-clamping was necessary for 11/66 patients (16.6%). In these symptomatic patients, the ipsilateral percent decrease of the MCAV was more than 70% in 8 out of 11 cases (72.7%). In asymptomatic patients, without shunt insertion, the average decrease of MCAV was less than 50%.

**Conclusions:**

Neurological symptoms referring to cerebral ischemia may be superior to TCD monitoring of cerebral blood flow for detecting the necessity of a shunt. Regional anesthesia enables reliable, symptom-based monitoring of CEAs.

Carotid endarterectomies (CEAs) are performed as stroke preventive measures based on the demonstrated benefits observed during symptomatic and asymptomatic CEA trials [1,2]. Both local and general anesthesia can be provided for the procedure. General anesthesia has the drawback of monitoring needs in the intraoperative setting. The most often used techniques for this purpose are as follows: stump pressure measurements, transcranial Doppler (TCD) monitoring, electroencephalography, somatosensory evoked potentials, and near-infrared spectroscopy. Some of these techniques have low sensitivity to detect intraoperative ischemia, while others are operator dependent, are influenced by the anesthetics used, or have some technical limitations [3]. When performed under the use of general anesthesia to make clinical decisions on a routine or selective shunting, no method of monitoring in selective shunting has been shown to produce better outcomes [4]. The outcomes of CEA are similar when choosing routine or selective shunting. It has to be noted that using a selective shunting strategy, intraoperatively indicated shunting determined an increased stroke rate, likely due to intraoperative hypoperfusion [5]. A recent meta-analysis concluded that, based on the available data, none of the neuromonitoring methods can be suggested as more preferable when selective shunting is applied [6].

Although a recent Cochrane review demonstrated no differences between local and general anesthesia in the 30-day incidence of stroke and death [7], some clinicians prefer regional techniques because it is believed that they reduce the incidence of inappropriate shunt insertion and provide more cardiovascular stability [8]. Intraoperative neurological deficit may occur in 7–20% of patients undergoing CEAs in local anesthesia [9]. Recently, it has been shown that postoperative complications are more frequent and 30-day stroke rate is significantly higher in patients with an intraoperative neurologic deficit [10,11]. Carotid shunting has been described to reduce the risk of perioperative stroke due to hypoperfusion [12].

TCD monitoring using fixed probes is a useful tool for cerebral blood flow monitoring during CEA. It enables continuous monitoring of blood flow velocity in the middle cerebral artery during carotid surgery and can detect emboli during the procedure. Therefore, in the past decades, it was widely used for intraoperative monitoring of CEAs under general anesthesia [13]. However, data on cerebral hemodynamic changes during CEAs under locoregional anesthesia are scarce [14,15]. Given this, the aim of the study was to perform intraoperative TCD monitoring for CEAs under intermediate plexus block to describe cerebral hemodynamic changes during different phases of the procedure. In addition, we wanted to see whether cerebral blood flow velocities are different in patients in whom neurological symptoms appeared during cross-clamping compared to intraoperatively nonsymptomatic patients. The main purpose of the study was to test the value of the TCD monitoring in detecting patients at higher risk to develop ischemic neurological symptoms during CEAs after cross-clamping.

## 1 Patients and methods

Patients with unilateral hemodynamically significant carotid stenosis scheduled for elective endarterectomies at the Department of Surgery University of Debrecen entered the study.

Local anesthesia was performed under ultrasound guidance using the L12-4s linear probe of the TE7 Ultrasound System (Shenzhen Mindray Bio-Medical Electronics Co., Ltd., Nanshan, Shenzhen, China). A 22 G, 50 mm long regional block needle (Vygon Echoplex, Ecouen, France) was used. The appropriate point for the puncture was chosen by tracking the brachial plexus course, starting from the supraclavicular region to the area where the brachial plexus elements disappear. The needle was inserted perpendicularly to the skin and posteriorly to the sternocleidomastoid muscle, just below the superficial cervical fascia and the investing layer of the deep cervical fascia. Twenty milliliters of Ropivacaine (Naropin, Aspen Pharma Ltd., Dublin, Ireland) in a concentration of 375 μg/mL was injected within 5 min under ultrasound control.

Bilateral TCD measurements were performed using the Rimed Digilite TCD sonography (Rimed Ltd., Israel). The temporal window was used for insonation of the middle cerebral artery at 45–55 mm depth, depending on the best signal. A fixed probe was used to register systolic, diastolic, and mean blood flow velocities (MCAVs) throughout the procedure. MCAVs and their percent changes during the procedure were taken into account for further analysis. MCAVs were registered at different time points during the procedure as follows: (1) resting state, before regional block, (2) after block, before incision, (3) before cross-clamp, (4) after cross-clamp, (5) 5 min after cross-clamp, (6) 10 min after cross-clamp, (7) after declamping, and (8) during the postoperative period (4–6 h).

Patients were divided into two groups based on eventual clinical symptoms during cross-clamping. These symptoms were as follows: contralateral numbness or paresis of the extremities, facial palsy, aphasia, and loss of consciousness. In any cases of neurological worsening, a shunt insertion was performed by the operating neurosurgeon.

### 1.1 Statistical analysis

Middle cerebral blood flow velocities were analyzed. We also calculated the percent change of the MCAV during the procedure and compared them on operated and non-operated sides. After performing a normality test and curve analysis using skewness and distribution values, data are reported as means ± standard deviations or medians and 25–75% interquartile ranges (IQRs), as appropriate. Comparisons were performed using *t*-tests and Kruskal–Wallis tests as appropriate. For the comparison of categorical values, *χ* ^2^ tests were applied. Differences were considered significant if *p* < 0.05.


**Ethical approval:** The research related to human use has been complied with all the relevant national regulations, institutional policies, and in accordance the tenets of the Helsinki Declaration, and has been approved by the authors’ institutional review board or equivalent committee. The trial was registered in the ClinicalTrials.gov registry under the number NCT02665104. Investigations were approved by the institutional ethics board of the university (registration number: DE RKEB/IKEB:4364/2015).
**Informed consent:** Informed consent has been obtained from all individuals included in this study.

## 2 Results

Eighty patients were checked for eligibility, but during the preoperative TCD measurements, 14 were not included because of poor transtemporal acoustic windows. Sixty-six patients were included, among them 43 were males and 23 were females. During the CEA procedure, 55 patients were considered asymptomatic and 11 symptomatic based on their clinical symptoms. Their confounding factors are summarized in Table 1. No significant differences were found between the ages of patients and cardiovascular risk factors among the groups. There were 7 patients with ASA II and 59 with ASA III grading. One early (<48 h) ipsilateral ischemic stroke occurred in both groups during the hospitalization period.

**Table 1:** Clinical characteristics of asymptomatic and symptomatic patients

	Asymptomatic (*n* = 55)	Symptomatic (*n* = 11)	*p*-value
Age (years)	65.8 ± 6.2	69.3 ± 5.8	0.09
Female/male	20/35	3/8	0.56
BMI	26.9 ± 4.4	28.1 ± 6.5	0.04
Hypertension (Y/N)	47/8	9/2	0.74
Diabetes (Y/N)	17/38	5/6	0.35
Smoking (Y/N)	19/36	4/7	0.90
Coronary artery disease (Y/N)	21/34	8/11	0.76
Peripheral arterial disease (Y/N)	16/39	5/6	0.28
Stroke in history (Y/N)	15/40	4/7	0.54
Ischemic lesions on brain CT (Y/N)	30/25	6/5	0.74

### 2.1 Cerebral blood flow velocities in asymptomatic patients during endarterectomies

**Figure 1:**
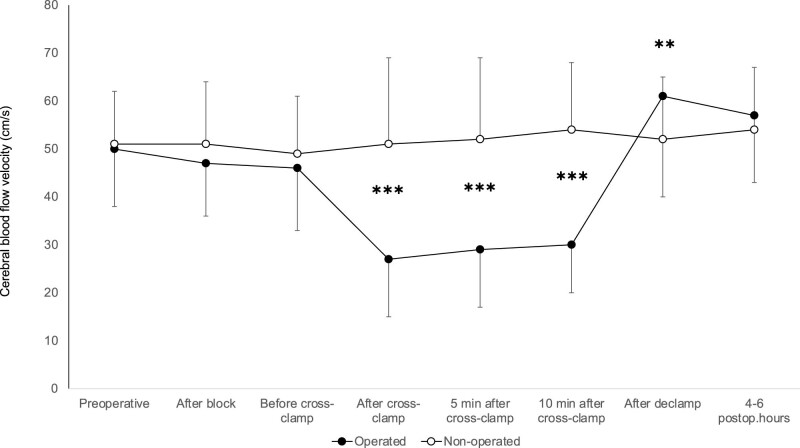
Middle cerebral artery mean blood flow velocity in asymptomatic patients. ✱✱✱ indicates *p* < 0.001.

MCAV changes showed a similar pattern in patients who did not experience any neurological symptoms during CEAs: a significant decrease of MCAV during cross-clamping and an increase of velocity on the operated side after declamping (Figure 1).

### 2.2 MCAVs in symptomatic patients

**Figure 2:**
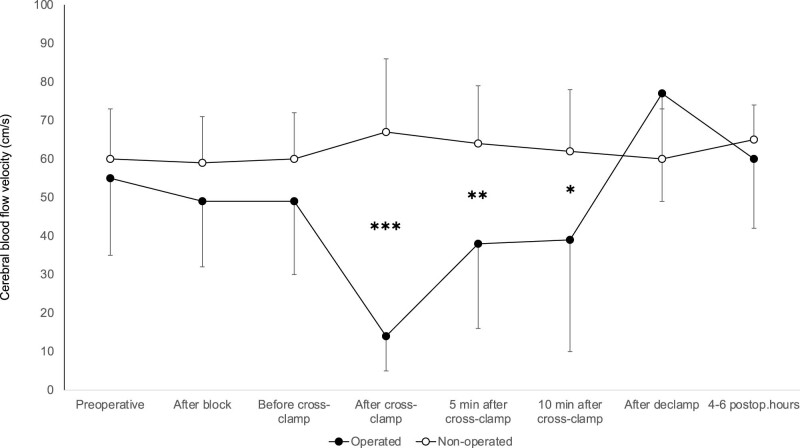
Middle cerebral artery mean blood flow velocity in symptomatic patients. ✱✱✱ indicates *p* < 0.001, ✱✱ indicates *p* < 0.01, ✱ indicates *p* < 0.05.

**Figure 3:**
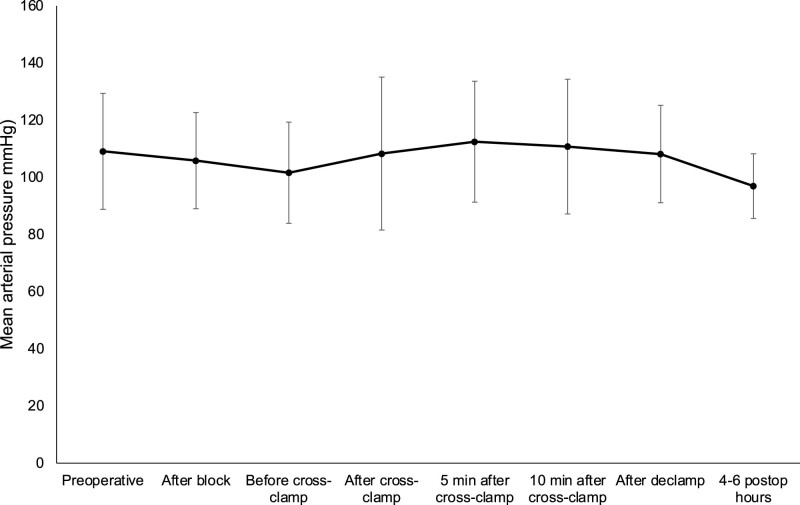
Mean arterial pressure during different phases of carotid surgery.

In patients, in whom neurological symptoms were observed after cross-clamping and shunt insertion was performed, the pattern of cerebral blood flow velocities during the procedure was different: When comparing the operated and non-operated sides, the decrease in MCAV was more pronounced on the operated side after cross-clamping than in asymptomatic patients, but after shunt insertion, the differences between the two sides were not so high. Furthermore, on the non-operated side, blood flow velocities slightly decreased. There was no significant difference between blood flow velocities of the operated and non-operated sides after declamping. Results are shown in Figure 2. The mean arterial blood pressures corresponding to the different phases of the study is depicted in Figure 3.

### 2.3 Comparison of percent changes of MCAV in asymptomatic and symptomatic patients

When comparing percent changes of the MCAV in symptomatic and nonsymptomatic patients, it was found that the % decrease of cerebral blood flow velocity during cross-clamping in symptomatic patients exceeded 70%, whereas it remained below 50% on average in asymptomatic cases. However, after insertion of the shunt in symptomatic patients, the percent decrease of blood flow velocity ipsilateral to surgery was lower in the symptomatic group than in the nonsymptomatic one. An increase in cerebral blood flow velocity on the operated side after declamping – indicating hyperperfusion – was somewhat higher in the symptomatic group than in the asymptomatic group (25 vs 17.6%, respectively). Results are summarized in Table 2.

**Table 2:** Percent changes of MCAV in asymptomatic and symptomatic patients on the operated and non-operated sides

	Asymptomatic patients (*n* = 55)			Symptomatic patients (*n* = 11)		
Phase	Operated side	Non-operated side	*p*-value	Operated side	Non-operated side	*p*-value
After block	−6.4 (−12.1/0)	0.0 (−7.8/7.7)	<0.01	−9.4 (−15.2/−2.2)	−7.5 (−9.3/4.8)	0.12
Before cross-clamp	−7.8 (−19.4/3.6)	3.1 (−10.4/4.5)	0.10	−12.5 (−25.9/−0.8)	−3.7 (−8.6/14.8)	0.07
After cross-clamp	−46.7 (−58.8/−34)	1.9 (−8.4/11.9)	<0.001	−71.0 (−85.2/−66.1)	11.0 (0.2/27.3)	<0.001
5 min after cross-clamp	−42.7 (−56.2/−28.4)	2.4 (−7.6/14.6)	<0.001	−23.6 (−54.3/−14.9)	−1.92 (−6.9/26.3)	<0.001
10 min after cross-clamp	−36.8 (−54.8/−25.7)	4.1 (−3.9/14.1)	<0.001	−31.3 (−53.2/−20.6)	1.9 (−9.1/11.1)	<0.01
After declamp	16.7 (−2.3/47.2)	2.1 (−6.6/10.9)	<0.001	25.0 (14.8/72.7)	0 (−11.6/7.8)	<0.01
4–6 postop. hours	11.5 (3.1/26.7)	5.3 (−1.9/16.1)	=0.052	10.9 (2.6/25.9)	5.8 (1.7–15.6)	0.74

Medians and 25–75% IQRs are shown.

## 3 Discussion

In the present study, we found that patients who needed shunt insertion because of neurological symptoms during cross-clamping showed a more than 70% decrease in the middle cerebral artery blood flow velocity ipsilateral to the CEA. After shunt insertion, this decrease was less intense than in asymptomatic patients without shunting. To our best knowledge, there are only a few studies that assessed cerebral blood flow velocities during regional anesthesia in patients undergoing carotid stenosis throughout the entire procedure.

In the past decades, an intensive debate was observed regarding the ideal method for detecting the need for shunt insertion during carotid surgeries. In a study of CEAs performed under regional anesthesia, McCarthy et al. described that shunt insertion based on the deterioration of neurological symptoms after cross-clamping was necessary in 12% of the cases [14]. This rate is somewhat lower than ours (11/66; 16.6%), but shunting rates based on the results of neuromonitoring during general anesthesia may be as high as 25% [16]. We have to comment on our cohort’s relatively high (16.7%) shunt rate. Shunting in our study was based on the appearance of neurological symptoms after cross-clamp. In a recent study using regional anesthesia, in 31.6% of the patients with post-clamping neurological symptoms, patch endarterectomy with shunting was used [17]. It has to be noted that the annual ischemic stroke rate in Hungary is 40/10,000 inhabitants/year [18] indicating largely cardiovascular affected population. In ischemic stroke patients with severe ICA occlusive disease, the odds ratios of a nonfunctional anterior and a nonfunctional posterior collateral pathway were found 7.33 (95% confidence interval [CI] = 1.19–76.52) and 3.00 (95% CI = 0.77–12.04), respectively, in a previous study performed in the same population [19]. As the patency of the Willisian collaterals play an important role in the decision making process about need for shunting [20], this higher incidence may be ascribed to a more severe intracranial vascular disease state in our patients resulting in nonfunctional intracranial collaterals during cross-clamping.

In line with our results, several authors reported a 50–70% decrease in blood flow velocity after cross-clamping during general anesthesia [16,21]. Continuous TCD monitoring using regional anesthesia performed showed that patients who tolerated carotid artery clamping had significantly higher MCAVs than patients who did not (26.2 ± 8.5 vs 1.8 ± 1.1 cm/s) [15]. In their comparative study, Moritz et al. proved that relative changes are more accurate for the diagnosis of cerebral ischemia than absolute blood flow velocity values. They reported a 50% reduction in MCA velocity, providing 100% sensitivity and 86% specificity. When they intended to exclude false-positive results (100% specificity), the corresponding reduction in MCA velocity was 70% [16]. Cao et al. also demonstrated that a greater than 70% reduction was the best TCD criterion for detecting the need for a shunt. In their series, TCD had a sensitivity of 83%, a specificity of 96%, a 71% positive, and a 98% negative predictive value [22]. These observations are in line with ours: in patients who developed symptoms during carotid cross-clamping, the ipsilateral percent decrease of the cerebral blood flow velocity was more than 70% in 8 out of 11 cases (72.7% sensitivity), the specificity was 93.1%, and the accuracy of TCD was 89.23%. Drawing the threshold of MCA velocity decrease at 50% after cross-clamping resulted in 90.1% sensitivity and 59.6% specificity, respectively, with an accuracy rate of 64.62%.

These results along with the previous observations may lead clinicians to reconsider the value and the role of TCD in monitoring CEAs performed under general anesthesia. TCD has the advantage of its ability to noninvasively monitor changes in cerebral blood flow velocities on both sides and detect embolizations occurring during the procedure. However, as indicated in several previous reports, its sensitivity and specificity for detecting the need for a shunt in CEAs performed under general anesthesia may be restricted [14,16]. Additional use of TCD might be that, in combination with carotid compression, the technique may be useful for preoperative assessment of the patency of the Willisian collaterals. This method is preoperative modeling of the effect of cross-clamping occurring during carotid surgery. In a recent study, it has been proven that an incomplete circle of Willis may be a risk factor for neurological events during endarterectomies [23]. TCD combined with carotid compression test enables a functional assessment of the Willisian collaterals [19] before CEAs under general anesthesia and hence may delineate the need for a shunt. However, due to the eventually occurring embolizations during compression tests, its use is very restricted and may be considered unethical.

We have to mention the limitations of the present study. Although the inclusion of 80 patients was planned, we had to exclude 14 patients due to inappropriate temporal acoustic windows. In these patients, TCD monitoring could not be performed. It is worth mentioning that among these 14 patients, an additional 3 patients developed neurological symptoms during cross-clamping. Another limitation might be that we only included patients with unilateral hemodynamically significant carotid stenosis. This was decided because a contralateral, hemodynamically significant stenosis makes hemodynamic changes largely intracranial collateral dependent during cross-clamping. We intended to assess hemodynamic changes ipsilateral to surgery in an isolated fashion. Third, we assessed cerebral blood flow velocities in the middle cerebral arteries and we do not have any information on hemodynamic changes in the anterior and posterior cerebral arteries or eventual steal phenomena occurring during cross-clamping.

In conclusion our results indicate that neurological symptoms referring the cerebral ischemia may be superior to TCD monitoring of cerebral blood flow for detecting the necessity of a shunt. Regional anesthesia enables reliable, symptom-based monitoring of CEAs.
